# The abrogation of the HOXB7/PBX2 complex induces apoptosis in melanoma through the miR-221&222-c-FOS pathway

**DOI:** 10.1002/ijc.28097

**Published:** 2013-02-07

**Authors:** M Cristina Errico, Federica Felicetti, Lisabianca Bottero, Gianfranco Mattia, Alessandra Boe, Nadia Felli, Marina Petrini, Maria Bellenghi, Hardev S Pandha, Marco Calvaruso, Claudio Tripodo, Mario P Colombo, Richard Morgan, Alessandra Carè

**Affiliations:** 1 Department of Hematology, Oncology, and Molecular Medicine, Istituto Superiore SanitàRome, Italy; 2 Faculty of Health and Medical Sciences, University of SurreyGuildford, United Kingdom; 3 Department of Health Sciences, University of PalermoItaly; 4 Department of Experimental Oncology and Molecular Medicine, Fondazione IRCCS Istituto Nazionale TumoriMilan, Italy

**Keywords:** HOXB7, PBX, microRNA, HXR9 peptide, melanoma

## Abstract

Cutaneous melanoma is the fastest increasing cancer worldwide. Although several molecular abnormalities have been associated with melanoma progression, the underlying mechanisms are still largely unknown and few targeted therapies are under evaluation. Here we show that the HOXB7/PBX2 dimer acts as a positive transcriptional regulator of the oncogenic microRNA-221 and -222. In addition, demonstrating c-FOS as a direct target of miR-221&222, we identify a HOXB7/PBX2→miR-221&222 →c-FOS regulatory link, whereby the abrogation of functional HOXB7/PBX2 dimers leads to reduced miR-221&222 transcription and elevated c-FOS expression with consequent cell death. Taking advantage of the treatment with the peptide HXR9, an antagonist of HOX/PBX dimerization, we recognize miR-221&222 as effectors of its action, in turn confirming the HXR9 efficacy in the treatment of human melanoma malignancy, whilst sparing normal human melanocytes. Our findings, besides suggesting the potential therapeutic of HXR9 or its derivatives in malignant melanoma, suggest the disruption of the HOXB7/PBX2 complexes, miR-221&222 inhibition or even better their combination, as innovative therapeutic approaches.

Malignant melanoma is the most aggressive form of skin cancer whose incidence has more than tripled in the white population during the last 20 years. Although surgical excision is mostly a definitive treatment at the early stages of the disease, at present standard treatments are ineffective after metastatic dissemination and patients with advanced disease have a grim prognosis, with a 5-year survival rate of less than 20%.^1^ The homeobox (*HOX*) genes are a family of homeodomain containing transcription factors that define the identity of cells and tissues during early development.^2^ Most cases of aberrant *HOX* gene expression include *HOX* genes that are normally silenced in adult cells and re-expressed in a wide variety of neoplasias suggesting the *HOX* family as another class of oncofetal genes.^3^ A number of studies have shown the contribution of some HOX genes in cancer. These include HOXA5 and HOXA10, playing tumor suppressor functions in breast carcinoma, or HOXC11 inducing S100β, an established marker of melanoma progression.^4^–^6^ HOXB7 has been reported as a master regulator in the oncogenic hierarchy.^7^ HOX proteins bind to DNA through a highly conserved 60 amino acid sequence called the homeodomain. The specificity and stability of HOX binding to DNA are achieved when it forms complexes with cofactors such as PBX and MEIS in humans. In previous studies, we demonstrated *HOXB7* constitutive expression in melanoma primary lesions and cell lines whereby it is able to bind to the promoter and activate the transcription of *bFGF*.^8^ Also, by using a dominant-negative PBX mutant (PBXNT), we showed that HOXB7 requires PBX as a co-factor for its oncogenic activity.^9^ The HOX/PBX binding interaction, mediated through a specific and highly conserved hydrophobic hexapeptide,^10^,^11^ strongly increases HOX/DNA affinity. Synthetic peptides mimicking this hexapeptide motif can interfere with HOX/PBX binding, including the small cell permeable peptide HXR9, which specifically antagonizes the interaction between HOX and PBX interfering with the binding of these proteins to the “HOX/PBX” DNA consensus sites. This disruption in turn triggers apoptosis in cancer cells both *in vitro* and *in vivo*.^12^ Considering the significant role played by HXR9 in renal, ovarian and non small cell lung cancer (NSCLC) as well as in murine B16 melanoma cells,^12^–^15^ we investigated the effects of this novel peptide in human melanoma cell lines.

**What's new?**

Cutaneous melanoma is the fastest increasing cancer worldwide. Although several molecular abnormalities have been associated with melanoma progression, the underlying mechanisms are still largely unknown. Here the authors show that the HOXB7/PBX2 complex is a new transcriptional activator of the oncogenic microRNA-221 and 222 in melanoma inducing tumor malignancy. The authors also identified c-FOS as a direct target of miR-221&222 whose repression causes reduced apoptosis. The abrogation of HOXB7 and/or miR-221&222 or the disruption of HOXB7/PBX2 dimers by the peptide HXR9 might thus represent novel molecular approaches for advanced melanoma, an aggressive neoplasm refractory to traditional therapies.

Accumulating evidence suggests that the family of regulatory RNAs, so called micro RNAs (miRs), plays an important role in various human cancers. MiRs are small non coding RNAs (21–25 nucleotides) that bind to partially complementary sites in the 3' untranslated regions of target genes regulating gene expression mostly at post-transcriptional level.^16^ Among the most dysregulated microRNAs implicated in cancer are miR-221 and miR-222, whose expression is highly upregulated in a variety of solid tumors, including melanoma.^17^,^18^ Besides being positive regulators of the cell cycle, miR-221&222 play an anti-apoptotic role as several of their mRNA targets are pro-apoptotic in nature. Thus, the increased expression of these two miRs leads to enhanced cell proliferation and survival.^19^ Based on our previous studies demonstrating the tumorigenic role of miR-221 and miR-222 in melanoma development and progression,^18^ here we demonstrate the transcriptional activating role of HOXB7/PBX2 complex on these two micro RNAs and, in turn, miR-221&222 direct targeting of *c-FOS*. These data were also confirmed by the HXR9 peptide, able to interfere with the HOX/PBX dimerization, as a HXR9-dependent down-regulation of both miR-221 and -222 followed by c-FOS and apoptosis induction was observed. The potential value of the HOXB7/PBX2→miR-221&222→c-FOS pathway suggests the disruption of the HOXB7/PBX2 complexes, miR-221&222 inhibition or their combination as innovative therapeutic approaches in melanoma.

## Material and Methods

### Cell lines culture and transduction

Most of the human melanoma cell lines used in the current study was stabilized from surgical specimens obtained from primary or metastatic tumors at Istituto Nazionale Tumori in Milan (Italy) (Supporting Information Table S1). Cell lines were characterized for growth in soft agar and, whenever possible, their metastatic potential was evaluated into athymic nude mice.^8^,^18^ The A375 cell line was from the American Type Tissue Collection (Rockville, MD) and its metastatic variant A375M^20^ was kindly provided by Dr. R. Giavazzi (Ist. M. Negri, Bergamo). The Mel888 cell line was kindly provided by Dr. R. Morgan (University of Surrey, Guildford, UK). Normal human epidermal melanocytes from foreskin were obtained from Promocell (Heidelberg, Germany).

The biopsy melanoma specimens used in this study were obtained from the archives of the Human Pathology Section, University of Palermo. Five cutaneous primary and five lymph nodal metastatic samples were analyzed. Sampling and handling of human tissue material was carried out in accordance with the ethical principle of the Declaration of Helsinki.

The *HOXB7* cDNA encompassing its complete coding sequence was cloned into the expression vector pSG5. The pSG5 empty vector was used as an internal control. Overexpression of miR-221&222 was obtained in melanoma cells by using a lentiviral vector system, as reported.^18^ pMCEF-BRAFV600E construct was kindly provided by Dr. R. Marais (Institute of Cancer Research, London, UK). “Controls” are always intended as empty vector-transduced cell lines.

### Small-interfering RNA

HOXB7 and PBX2 were specifically silenced using small interfering RNAs (IDT, Leuven, Belgium). Briefly, 24 hr after plating, cells were transfected using Fugene HD (Promega, Madison, WI) with Dsi-HOXB7 (three different sequences were utilized: HSC.RNAI.N004502.12.1, HSC.RNAI.N004502.12.2, and HSC.RNAI.N004502.12.3), Dsi-PBX2 (HSC.RNAI.N002586.12.1), or a Dsi-RNA scrambled control (Dsi-scr #64218602) (final concentration 100 nM). The levels of HOXB7 and PBX2 mRNA and protein were analyzed 48 hr after transfection by qReal-time PCR and western blot. When indicated, cycloheximide (CHX) (50 µg/mL) was added in Dsi-transfected A375M cells and total protein extracts analyzed by Western blot at the indicated time points. SiRNAs for c-FOS were obtained from Ambion Inc. from the Silencer Select siRNA range. A negative control siRNA was used from the Ambion Silencer siRNA Starter Kit (AM1640), and transfections were performed using the siPORT NeoFX/Opti-MEM I transfection reagents, also included in the kit, according to the manufacturer's instructions. Transfections were performed in a 96-well plate using 0.8 µL siSPORT neoFx and 9.2 µL Opti-MEM I per well.

### MicroRNA-221 and -222 silencing by antagomir treatment

Chemically modified antisense oligonucleotides (antagomiRs) have been used to inhibit miR-221 and/or -222 expression *in vitro*, as described.^21^

### Chromatin Immunoprecipitation (ChIP) Assay

Cells (5 × 10^6^) from A375M melanoma cell line were fixed in 1% formaldehyde for 10 min at room temperature. Cells were washed with ice cold 1× PBS, scraped in 1× PBS plus protease inhibitors and collected by centrifugation. Cell pellets, resuspended in cell lysis buffer (50 mM Tris-HCl pH 8.0, 10 mM EDTA, 1% SDS) in presence of protease inhibitors, were sonicated. DNA-protein complexes were immunoprecipitated using 3 μg of anti-HOXB7 (Invitrogen Ltd., Paisley, Scotland UK), anti-PBX2 or, as an internal control, the unrelated anti-DVL-1 (Santa Cruz Biotechnology, Santa Cruz, CA). DNA-protein crosslinks were reversed by heating at 65°C overnight. The recovered DNAs were then PCR-amplified with the following primer set: DIR 5'-CAGCATACATGATTCCTTGTGA-3' and REV 5'-CTTTGGTGTTTGAGATGTTTGG-3', corresponding to (−352/+1) region and containing HOX/PBX BS1 at −255; DIR 5'-CCCACCAAGAGCTAACCACA-3' and REV 5'-TCGGACATGCAGCTATACCA-3' corresponding to (−1786/−1035) and containing HOX/PBX BS2 at −1390. Two negative control regions (−891/−698) and (−1066/−902) were also amplified. A positive control of amplification was carried out on input chromatin (preserved before immunoprecipitation) and a negative one on DVL-1 (unrelated)-immunoprecipitated chromatin. To confirm the specificity of the immunoprecipitated products, GAPDH PCRs were also run.

### Analysis of cell death and apoptosis detection

Cells received 40 µmol/L of HXR9, or the control peptide CXR9 for 2 hr, and were harvested for subsequent analyses. The assessment of cell viability was done using the MTS assay (Promega, Madison, WI) or the lactose dehydrogenase (LDH) cytotoxicity detection kit (Roche Molecular Biochemicals) according to the manufacturer's instructions. For the sub-G0 PI staining assay, the cells were incubated in a buffer containing 0.1% trisodium citrate, 9.65 mM NaCl, 0.1% NP40, 5 mg/ml PI, and 1 mg/ml RNase A, at room temperature for 30 min. Fluorescence emission was measured by flow cytometry. Cells with a sub-G0 content were identified as apoptotic. Apoptosis level was also determined by using the annexin V-FITC apoptosis detection kit as described by the manufacturer (R&D Systems, Abingdon, UK). The caspase inhibitor Z-VAD-fmk (R&D Systems, Abingdon, UK) was used at a concentration of 200 mM for 1 hr before CXR9 or HXR9 treatment.

### In vivo assay

All the animal experiments were conducted in accordance with the United Kingdom Co-ordinating Committee on Cancer Research (UKCCCR) guidelines for the Welfare of Animals in Experimental Neoplasia. The mice were kept in positive pressure isolators in 12 hr light/dark cycles and food and water were available *ad libitum*. Athymic nude mice were inoculated subcutaneously with a suspension of 2.5 × 10^6^ A375M cells in culture media (100 µL). Once tumors reached volumes of approximately 100 mm^3^, mice received an initial dose of 100 mg/kg CXR9 or HXR9 intratumorally. Each treatment group contained 10 mice. The mice were monitored carefully for signs of distress, including behavioral changes and weight loss.

### Statistical analysis

All data were presented as mean values ± standard errors (SE). Unless otherwise stated, results were representative of at least three independent experiments. Statistical analysis was performed using the *t*-test with *p* < 0.05 deemed statistically significant. *p* Values relative to HXR9-treated cells were always referred to CXR9-treated cells.

## Results

### HOXB7 and HOX cofactor expression in melanoma cell lines

**Figure 1:**
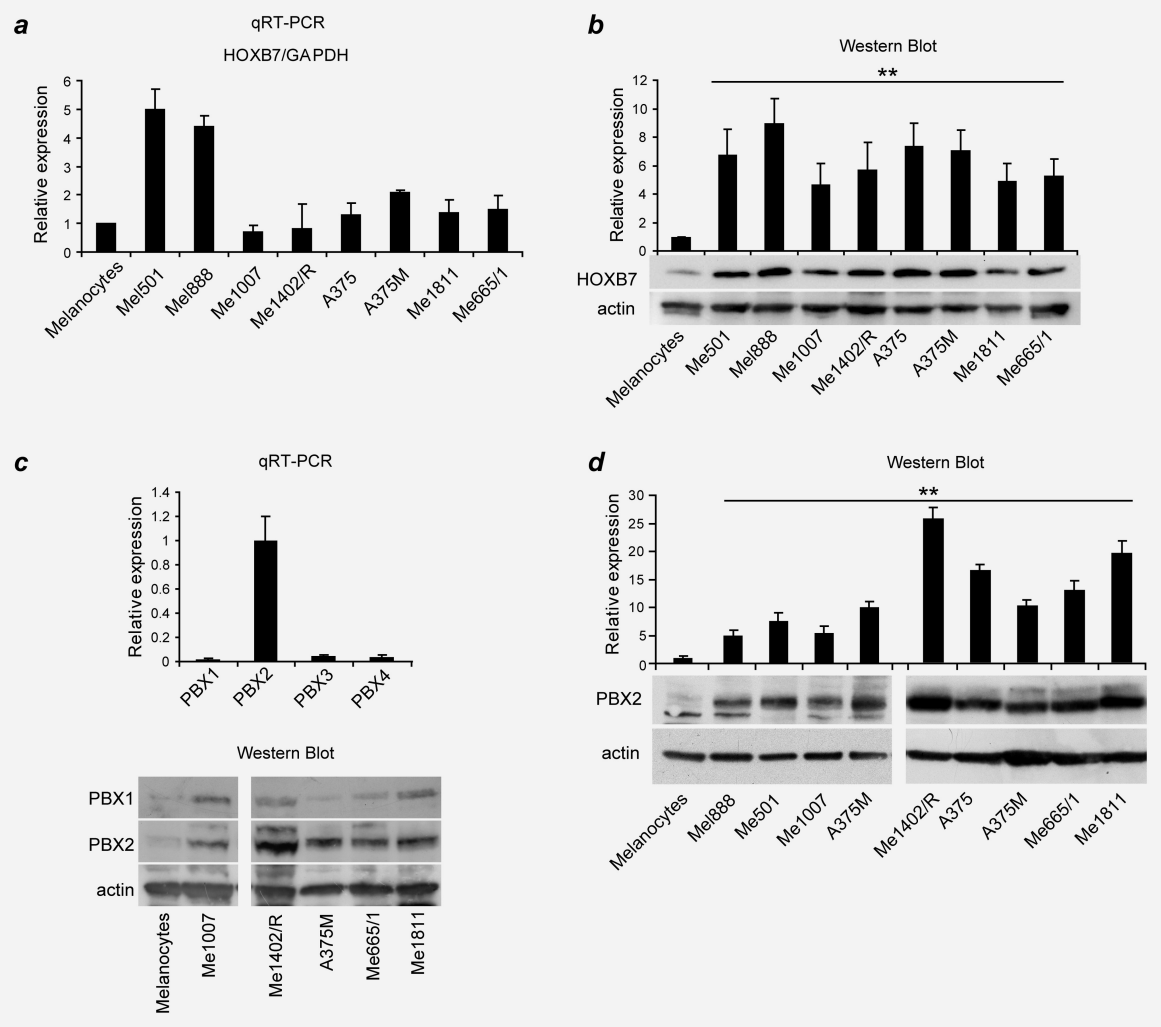
Expression analysis of *HOXB7* and *PBX2* in normal human melanocytes and melanoma cell lines. (*a*) qRT-PCR and (*b*) Western blot (WB) analyses of *HOXB7* with relative densitometric evaluation. (*c*) qRT-PCR of *PBX-1* to -*4* evaluated in the A375M melanoma cell line (top), WB analysis of PBX1 and PBX2 in melanocytes and some representative melanoma cell lines (bottom). (*d*) WB and relative densitometric analysis of PBX2. GAPDH and actin are the internal controls. **p < 0.001.

We previously reported the constitutive expression of *HOXB7* in both melanoma primary lesions and cell lines.^8^ Here we have confirmed and extended the expression pattern of *HOXB7* performing qRT-PCR and western blot analyses on normal human epidermal melanocytes from foreskin and on a panel of melanoma cell lines derived from tumors at different stages of progression (Figs. 1*a* and 1*b*) (Supporting Information Table S1). Although we found *HOXB7* mRNA expression in melanocytes and in all the analyzed melanomas (Fig. 1*a*), HOXB7 protein showed a statistically significant higher expression in melanoma cell lines compared to the normal counterpart (Fig. 1*b*). In view of the requirement of HOX cofactors, such as PBX proteins, for HOXB7 oncogenic activity,^9^ we measured the levels of PBX1 and PBX2 proteins in melanoma cell lines, representative of early and advanced stages, showing that PBX2 protein was more abundant than PBX1 (Fig. 1*c*, bottom). The analysis of the *PBX* family mRNAs (PBX1 to 4) in the metastatic melanoma cell line A375M confirmed *PBX2* principal expression (Fig. 1*c*, top). Finally, western blot analysis showed a significant higher level of PBX2 in melanoma cell lines respect to normal melanocytes (Fig. 1*d*).

**Figure 2:**
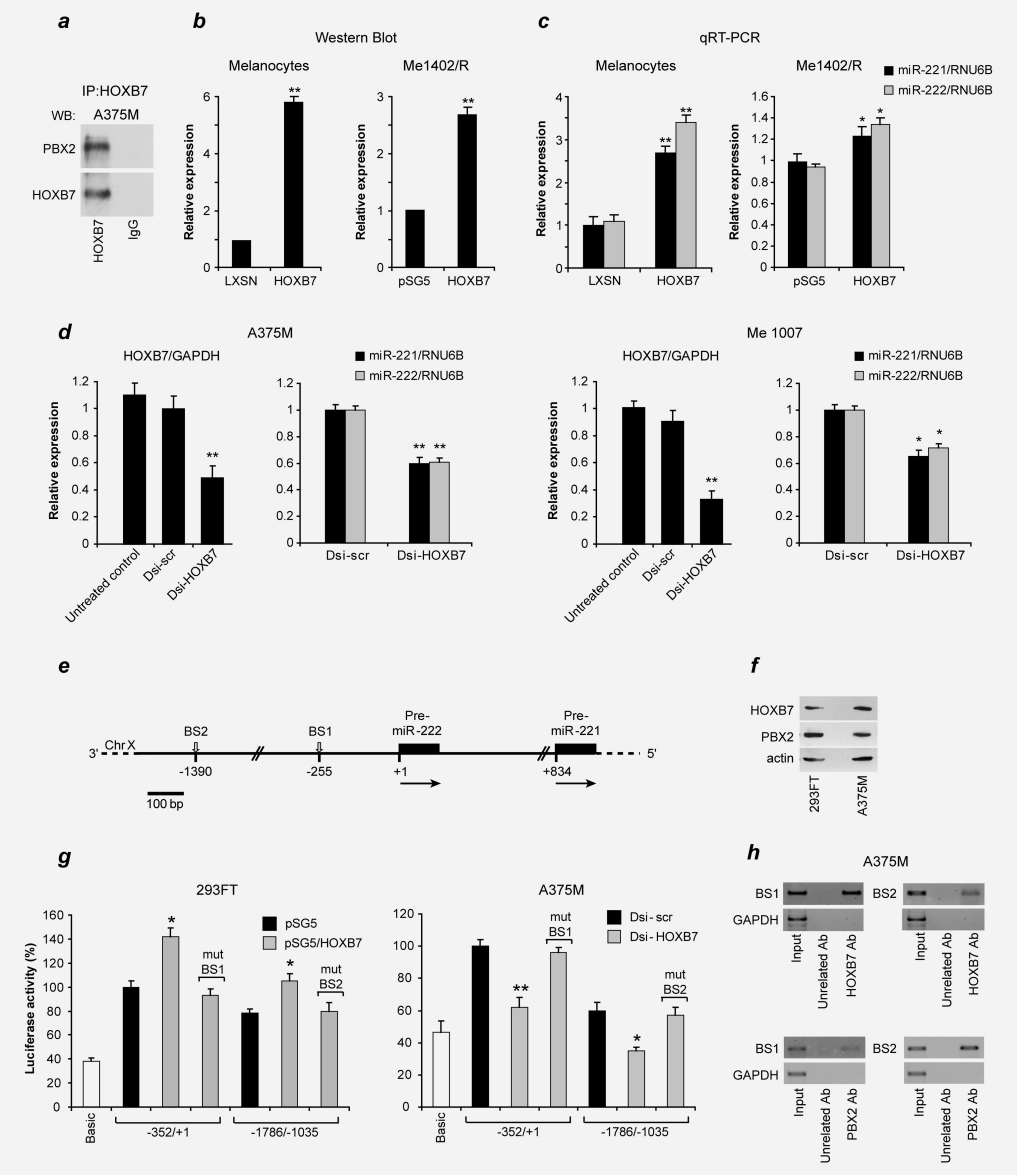
The HOXB7/PBX2 complex up-regulates miR-221&222. (*a*) Coimmunopreciptation analysis of PBX2 and HOXB7 in A375M cell lysates. Proteins were immunoprecipitated with the HOXB7 Ab and immunoblotted with HOXB7 and PBX2 Abs. (*b*) WB analysis of HOXB7 enforced expression in normal human melanocytes and in Me1402/R melanoma cell line. LXSN and pSG5 are the empty vector controls. (*c*) qRT-PCR of miR-221&222 normalized on *RNU6B* in the same cells as in *(b)*. (*d*) qRT-PCR evaluation of *HOXB7* and miR-221&222 in Dsi-HOXB7-transfected A375M *(*left*)* and Me1007 *(*right*)* cell lines. (*e*) Schematic depiction of the genomic region upstream to pre–miR-221&222. BS1 and BS2 indicate the HOX/PBX binding sites. (*f*) WB analysis of endogenous HOXB7 and PBX2. (g) Promoter luciferase assays performed in the 293FT and A375M cells transfected with either HOXB7 expressing vector or a Dsi-HOXB7. As controls, empty vector, Dsi-scrambled and mutated binding sites were included. (*h*) Chromatin immunoprecipitation assay performed in A375M cells and subsequently analyzed by semiquantitative PCR. *GAPDH* and *RNU6B* were used for normalization. *p < 0.05. **p < 0.01. *mut*, mutated.

### The HOXB7/PBX2 complex regulates miR-221&222 transcription

It is well known that the interaction between HOX proteins and their PBX cofactors is dependent on a short stretch of highly conserved amino acids in the amino terminal of the HOX protein, identified as the hexapeptide sequence.^10^,^11^ Here we investigated whether a direct HOXB7/PBX2 interaction does exist and if this heterodimeric binding is functionally required for melanoma induction and/or progression. To this end, the putative HOXB7/PBX2 complex was co-immunoprecipitated by using an anti-HOXB7 specific antibody and thereafter evaluated by western blot with an anti-PBX2. As shown in Figure 2*a*, results confirmed the existence of HOXB7/PBX2 heterodimers in the A375M metastatic cell line.

Previous microRNA expression profiles, confirmed by qRT-PCR analysis, had shown that *HOXB7* retroviral transduction was sufficient to induce miR-221 and -222 up to three and five-fold, respectively, in a model of SkBr3 mammary carcinoma cell line (our unpublished results). The increase of both microRNAs upon *HOXB7* expression in these cells prompted us to look for the possible involvement of HOXB7 and PBX2 in miR-221&222 positive regulation. To demonstrate that the HOXB7/PBX2 dimer can activate the transcription of miR-221&222 in melanoma, we selected the Me1402/R cell line for *HOXB7* transient over-expression (Fig. 2*b*, right). qRT-PCR analysis demonstrated that 48 hr after transfection, the endogenous levels of miR-221 and miR-222 displayed a small, but consistent 20 to 30% increase as compared with empty vector (pSG5)-transfected control cells (Fig. 2*c*, right*)*. In addition, we confirmed the HOXB7-dependent induction of miR-221&222 in normal human melanocytes lentivirally infected with HOXB7. As shown, a three-fold induction of both microRNAs was obtained as a consequence of HOXB7 overexpression (Figs. 2*b*, left and 2*c*, left).

The specificity of this HOXB7/PBX2*-*dependent activation was further sustained by miR-221&222 downregulation when *HOXB7* or *PBX2* were knocked-down. Results were always obtained by comparing Dsi-HOXB7 or Dsi-PBX2-treated cells with Dsi-scrambled (Dsi-scr) and/or untreated controls (Fig. 2*d* and Supporting Information Fig. S1a). In order to understand the molecular bases of HOXB7 action on the regulation of these microRNAs, we looked for HOX/PBX binding sites (BS) in the ∼1.8 kb sequence upstream to pre-miR-221&222. Bioinformatic analysis using MatInspector software (http://www.genomatix.de) indicated the presence of two canonical HOX/PBX consensus sequences (TAAT/TGAT) located in the promoter region at position −255 and −1390 (Fig. 2*e*). To test whether these putative BSs were truly functional, we performed a series of promoter luciferase assays by utilizing the highly transfectable 293FT and the metastatic melanoma A375M cell lines, endogenously expressing both HOXB7 and PBX2 (Fig. 2*f*). Sequences comprised between −1786 and +1 nt and containing the HOX/PBX BSs were cloned in a promoter-less pGL vector and cotransfected with either a *HOXB7*-containing expression vector or a Dsi-HOXB7. In the presence of the genomic fragments (−352/+1) or (−1786/−1035), *HOXB7* induced a 30% increase, whereas its silencing produced a 40% decrease of the luciferase activity, thus indicating a HOXB7-dependent transcription. Furthermore, point mutations, inserted in the core binding sequences for HOX/PBX, restored luciferase to the background levels, demonstrating that the binding sites identified were specifically responsible for most of the HOXB7-induced activation (Fig. 2*g*).

Finally, we demonstrated by chromatin immunoprecipitation (ChIP) assays in the A375M cells the *in vivo* direct interaction of HOXB7 and PBX2 proteins with the putative cis-regulatory elements present on miR-221&222 promoter: PCR amplifications of unsheared input genomic DNA, anti-HOXB7 or anti-PBX2 antibody-mediated reactions revealed a significant HOXB7 and PBX2 binding to these BSs (BS1 and BS2) compared with a negative control obtained by immunoprecipitation with an irrelevant antibody (Fig. 2*h*).

### MiR-221&222 directly target c-FOS

The upregulation of c-FOS has been reported as a key event in apoptosis induction in cancer cells.^12^ We here searched for the option of a miR-221&222 dependent regulation of c-FOS.

**Figure 3:**
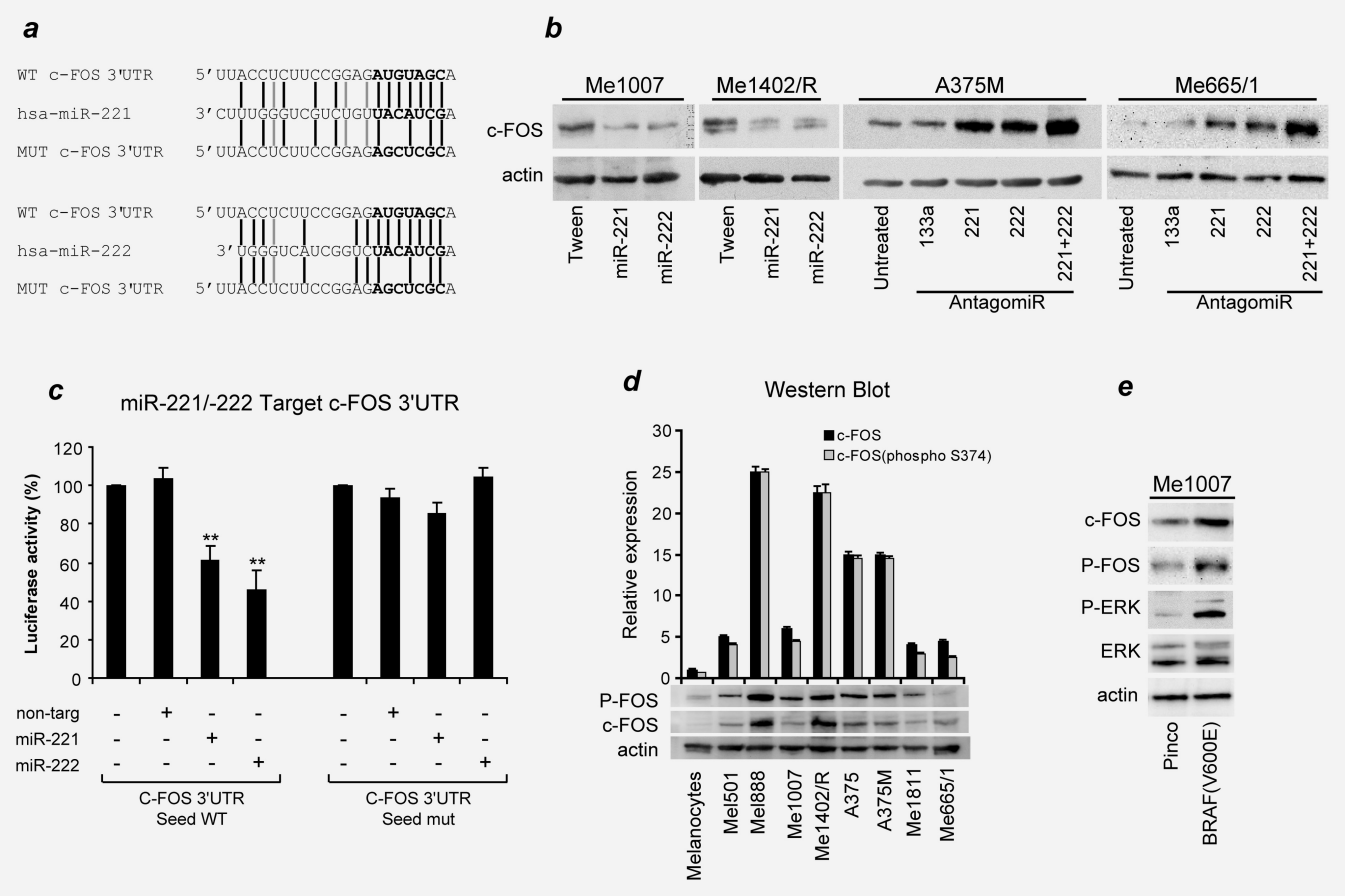
c-FOS targeting by miR-221&222. (*a*) Nucleotide pairing between the *c-FOS* 3'UTR and miR-221&222 is shown by bars. In the seed sequence (bold font), mutated nucleotides are shown as mismatched. (*b*) Representative WB analysis of c-FOS in Me1007 and Me1402/R control (Tween vector) *versus* miR-221- or 222-infected cells *(*left*)* and antagomiR-221 and/or 222-transfected A375M and Me665/1 melanoma cells *(*right*)*; antagomiR-133a represents a negative control. (*c*) Luciferase (LUC) reporter assay performed by cotransfecting miR-221 and/or miR-222 in the presence of the LUC reporter gene linked to *c-FOS* 3'UTR. As controls, mutated 3'UTR sequences and a non-targeting oligomer were also included. Representative WB of (*d*) PSer^374^-FOS (P-FOS) and c-FOS in normal human melanocytes and melanoma cell lines and (*e*) P-FOS, c-FOS, P-ERK1/2 and total ERK1/2 in BRAF^V600E^-transfected Me1007 cell line. **p < 0.01. *mut*, mutated.

Basing on bioinformatics analyses (http://www.targetscan.com), we found the presence of one conserved binding site for miR-221&222 in the 3'UTR of the c-FOS transcription factor (Fig. 3*a*). The negative regulation of c-FOS by miR-221&222 was confirmed by western blot analysis in miR-221&222-transduced Me1007 and Me1402/R melanoma cell lines (Fig. 3*b*, left). Notably, the opposite expression pattern was observed in the antagomiR-treated A375M and Me665/1 metastatic melanoma cell lines, where a significant induction of c-FOS was obtained by miR-221 and/or -222 abrogation (Fig. 3*b*, right). It is important to note that miR-221 and miR-222 appear to equally contribute to c-FOS regulation. To verify that c-FOS was directly targeted by miR-221&222, the 3'UTR region of *c-FOS*, containing either wild type or mutated miR-221&222 binding sequences, was cloned downstream to the luciferase open reading frame. The presence of wild-type ‘‘seed’’ caused a 40% and 50% inhibition of the luciferase activity in presence of cotransfected miR-221 and -222, respectively. In contrast, the luciferase level of the mutated 3'UTR was unaffected by both miRs, thus confirming the specificity of miR-221&222 dependent targeting of *c-FOS* (Fig. 3*c*).

We then assessed the inverse correlation of miR-221&222 and c-FOS expressions by comparing their endogenous levels in normal melanocytes and melanoma cell lines. As miR-221&222 expressions are directly related with melanoma progression, being almost undetectable in normal human melanocytes and increasingly expressed throughout transformation process (Supporting Information Fig. S3a), c-FOS was expected to decline from primary to metastatic melanoma. Nonetheless at first sight the expression results did not show a clear opposite pattern of miR-221&222 and c-FOS (Fig. 3*d*), thus suggesting a more complex regulation. C-FOS is known to be stabilized by phosphorylation at serines 362 and 374 upon ERK1/2 activation downstream to the MAPK pathway, which is constitutively activated in most melanomas.^22^–^24^ Phospho-FOS (P^S374^-FOS) and c-FOS evaluation by western blot confirmed their variable levels, thus supporting the complex relationship between miR-221&222 expression levels and c-FOS phosphorylation and stability (Fig. 3*d*). Considering that metastatic melanomas are frequently characterized by BRAF ^V600E^ heterozygous mutation,^25^ we assessed its role on c-FOS stability by transiently transfecting BRAF ^V600E^ in the early primary Me1007 cell line, endogenously expressing wild-type BRAF. After transfection, we observed ERK1/2 activation, P^S374^-FOS induction and, as a consequence of this stabilizing phosphorylation, higher c-FOS levels with respect to the control (Fig. 3*e*). In view of this multifaceted regulation, the reduced expression of c-FOS detected in metastatic melanomas could rely on a balance between c-FOS stability and the high levels of miR-221&222.^18^

### HXR9 induces apoptosis in metastatic melanoma *in vitro* and *in vivo*

To further confirm the activating function of the HOXB7/PBX2 complex, we took advantage of the peptide HXR9. This designed peptide is a competitive inhibitor of the HOX/PBX dimerization which disrupts their interaction by mimicking the highly conserved hexapeptide in the HOX protein.^12^ In all the experiments, as an irrelevant control, we utilized CXR9, a peptide lacking the functional PBX binding site through a single amino acid substitution.

**Figure 4:**
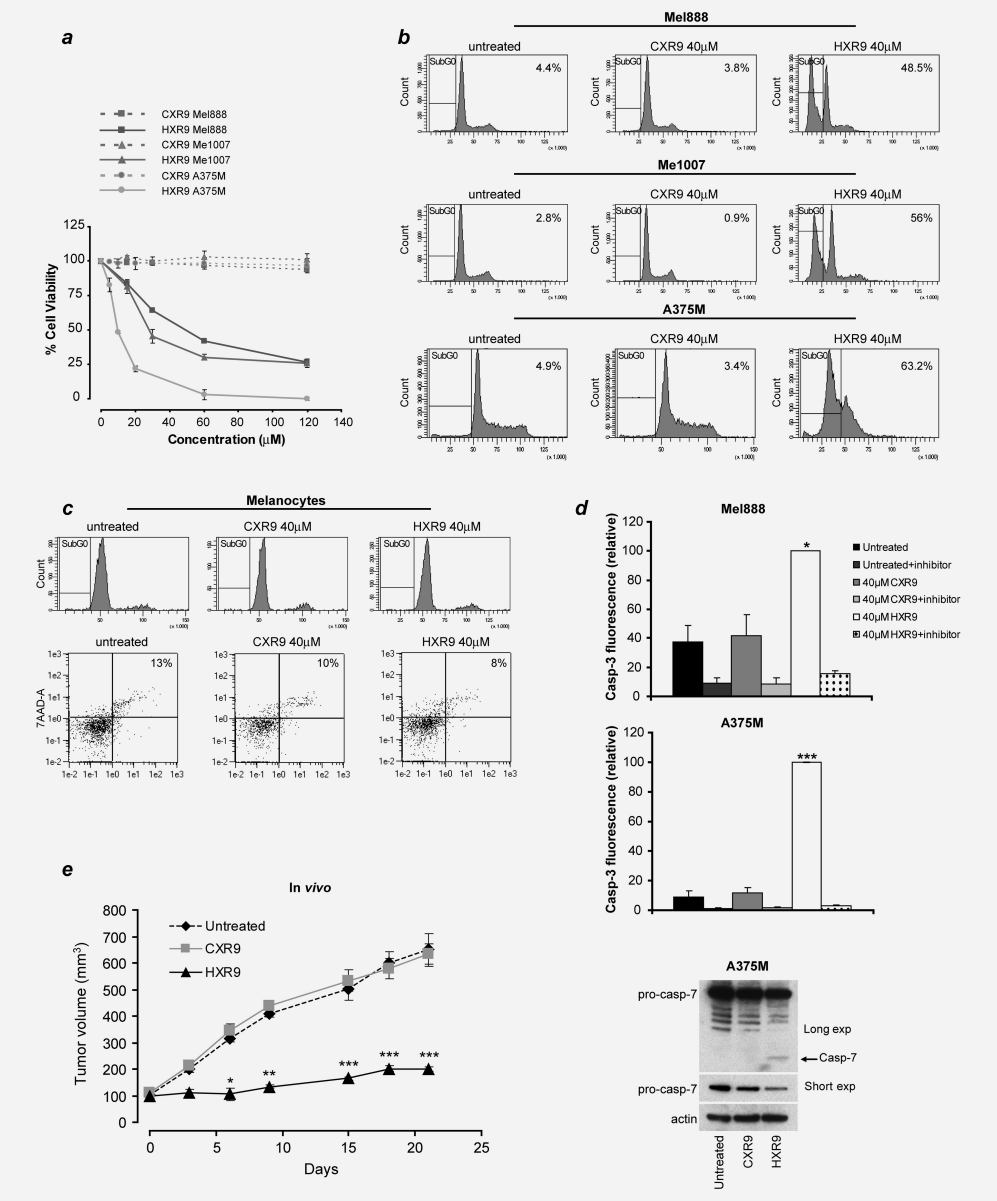
Toxicity and cell death evaluation after HXR9 treatment. (*a*) Viability of Mel888, Me1007 and A375M cells assessed with the MTS assay. The resulting IC_50s_ were 49 µM, 30 µM and 10 µM, respectively. (*b*) Representative apoptosis analysis of (b) PI-sub-G0 stained population of CXR9-or HXR9-treated melanoma cell lines and (*c*) PI-sub-G0 (top) or annexin V-FITC and 7AAD-A staining (bottom) of normal human melanocytes. (*d*) Detection of caspase-3 activity in Mel888 and A375M cells *(*top). Reversible aldehyde inhibitor was included as a control. WB of pro-caspase-7 and active caspase-7 in A375M (bottom). The longer exposure evidences the active form of caspase-7. (*e*) *In vivo* tumor growth into athymic nude mice of A375M derived tumor nodules either untreated or intratumorally treated with HXR9 or CXR9. *p < 0.05, **p < 0.01, ***p < 0.001.

At first we performed a series of experiments to confirm the HXR9 antineoplastic functions in a number of human melanoma cell lines. Looking for HXR9 cytotoxicity, the MTS assay revealed that HXR9 inhibited Mel888, Me1007 and A375M cell growth with IC_50s_ of 48, 30 and 10 µM, respectively, whilst the CXR9 control peptide had no discernible effects (Fig. 4*a*).

To evaluate the potential pro-apoptotic effects of HXR9, we used the propidium iodide staining checking for the sub-G0 accumulation of cells. Treatments of Mel888, Me1007 and A375M cells with different HXR9 concentrations (properly ranging from 5 to 120 µM, according to the each different IC_50_) resulted in a marked dose dependent increase of the sub-G0 population (Supporting Information Fig. S2). By comparing the results obtained with the 40 µM dose, we observed a HXR9 significant induction of apoptosis in all the treated cell lines, with a maximum of 63.2% in the A375M metastatic cell line (Fig. 4*b*). In order to see whether HXR9 would also induce apoptosis in normal cells, we performed both flow cytometry of sub-G0 cells and annexin V and 7AAD staining analyses on a population of normal human melanocytes. Interestingly, HXR9-treated melanocytes did not undergo any form of cell death, revealing absence of cytotoxic effects in non-tumorigenic cells (Fig. 4*c*).

To establish whether the cells where actually dying through an apoptotic pathway, we applied a direct assay for caspase-3 activity, an early indicator of apoptosis, using a fluorescently labeled caspase-3 substrate. HXR9 treatment resulted in a marked activation of caspase-3 in A375M and Mel888 cell lines when compared with untreated and CXR9-treated cells (Fig. 4*d*, top). A specific aldehyde inhibitor was utilized to confirm the specificity of fluorescence emission as a result of caspase-3 activity. In addition, western blot analysis showed a decreased amount of pro-caspase-7 as well as an increase of the active proteolytic fragment in HXR9-treated A375M *versus* controls (Fig. 4*d*, bottom). The A375M cells treated with the pan-caspase inhibitor z-VAD-fmk before HXR9 treatment, showed a 25% of protection from a HXR9-induced cell death (data not shown).

In order to evaluate whether HXR9 might be able to block melanoma cell growth also *in vivo*, we subcutaneously injected into athymic nude mice the A375M cells. Once tumors had reached 100 mm^3^, mice received a single intratumor dose of HXR9 or CXR9 and tumor growths were followed and compared over approximately 3 weeks. As shown (Fig. 4*e*), HXR9-treated tumors showed a significant degree of growth retardation compared to the control groups.

### HXR9 blocks the HOXB7/PBX2-miR-221&222-c-FOS pathway

**Figure 5:**
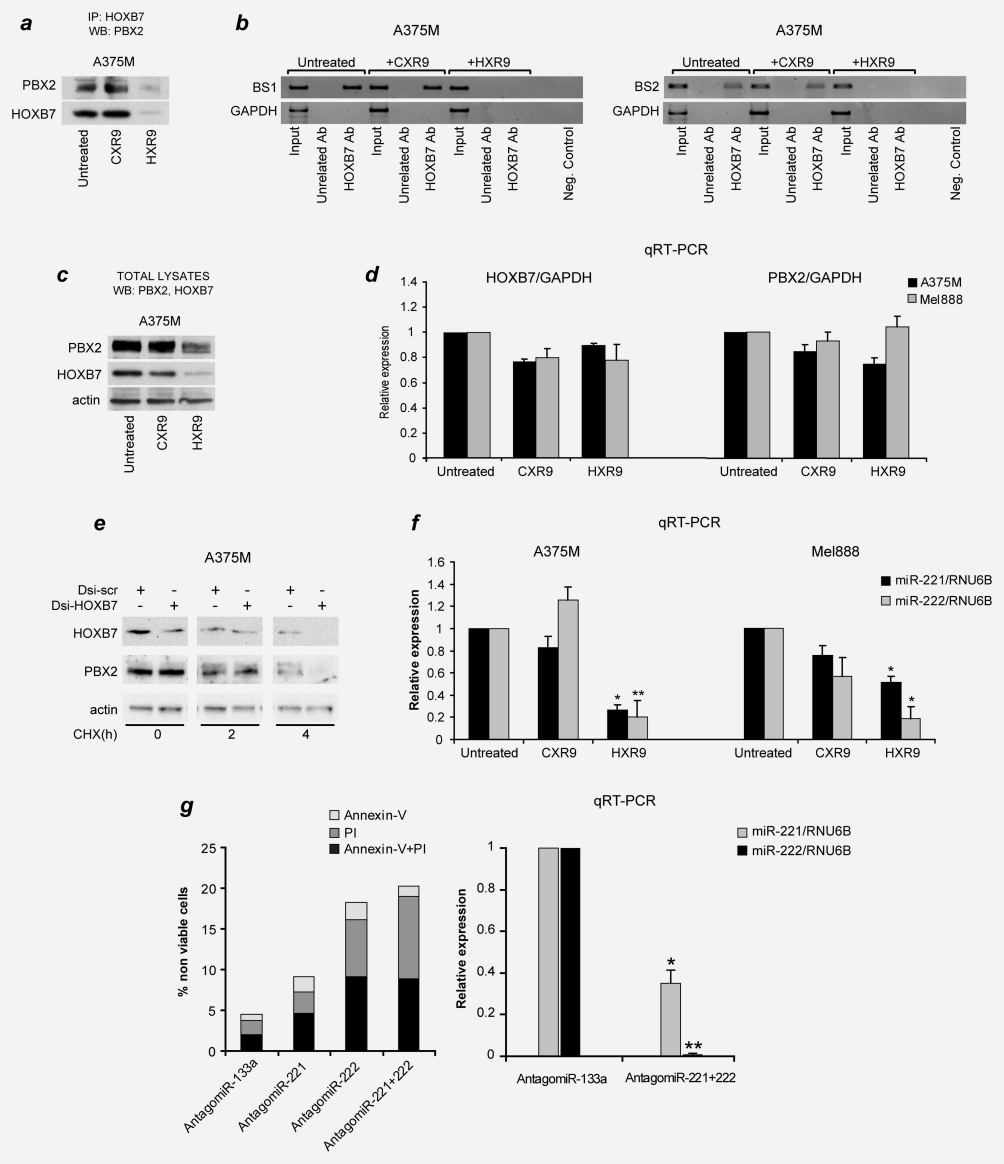
HXR9-dependent effects in A375M and Mel 888 melanomas. (*a*) Coimmunoprecipitation and (*b*) Chromatin immunoprecipitation assays performed in A375M cells treated with 40 µmol/L of HXR9 **or** CXR9. (*c)* WB analysis of PBX2 and HOXB7 in total cell lysates kept apart before co-immunoprecipitation. (*d*) qRT-PCR analysis of *HOXB7* and *PBX2* levels in A375M and Mel888 melanoma cell lines. (*e*) A375M cells transfected with either control Dsi-scr, or with Dsi-HOXB7 and treated with cycloheximide (CHX). HOXB7, PBX2 and actin protein levels were evaluated at the indicated time points. (*f*) Evaluation of miR-221 and miR-222 levels by qRT-PCR in HXR9-treated A375M and Mel 888 cells compared with CXR9 controls. (*g*) Flow cytometric evaluation of apoptotic cells in antagomiR-treated A375M cells *(*left*)*; miR-221&222 reduction confirmed by qRT-PCR *(*right*)*. Actin, *RNU6B* and *GAPDH* were used as internal controls. AntagomiR-133a was used as a negative control. *p < 0.05, **p < 0.01.

The competence of the HOXB7/PBX2-miR-221&222-c-FOS pathway was then verified in presence of either the HXR9 or the CXR9 peptides. The A375M cell line treated with the HXR9 peptide was then analysed by co-immunoprecipitation and ChIP. The HXR9 treatment totally abrogated the HOXB7 binding with PBX2 and, in turn, with DNA, endorsing the higher affinity of the HOX/PBX complexes for DNA (Figs. 5*a* and 5*b*). Co-immunoprecipitation studies, performed with the anti-HOXB7 antibody, allowed the recovery of PBX2 in untreated and CXR9, but not in HXR9-treated cells, confirming that HXR9 does indeed block the interaction between PBX2 and HOXB7 (Fig. 5*a*). Accordingly, chromatin immunoprecipitation (ChIP) assays in the A375M metastatic cells treated with the HXR9 peptide totally abrogated the HOXB7 or PBX2 binding to the analyzed BS1 and BS2. As a negative control we also run this immunoprecipitation with an unrelated irrelevant antibody (Fig. 5*b* and Supporting Information Fig. S1b). In addition the specificity of our results and the absence of other HOX/PBX binding sites was confirmed through the analysis of two negative control regions, (−891/−698) and (−1066/−902) (Supporting Information Fig. S1c).

Unexpectedly, the analysis of HOXB7 and PBX2, evaluated by western blot of total cell lysates kept apart before immunoprecipitation, showed a HXR9-dependent decrease of both HOXB7 and PBX2 expression levels (Fig. 5*c*). As the qRT-PCR analysis performed on Mel888 and A375M cell lines, as representative primary and metastatic melanomas, did not show any significant modulation of both mRNAs in response to HXR9 (Fig. 5*d*), we hypothesized a post-transcriptional regulation of HOXB7 as well as PBX2. Specifically, we looked for the option of HOX/PBX dimers being more stable than monomers. To this end, we silenced either HOXB7 or PBX2 in the A375M cells, in turn adding the protein synthesis inhibitor cycloheximide (CHX) in the differently silenced cells (*i.e*. Dsi-scr, Dsi-HOXB7, Dsi-PBX2). Interestingly, a reciprocal shortening of the protein half-lives was clearly visible either in Dsi-HOXB7- or Dsi-PBX2-transfected cells (*i.e*. HOXB7 downregulation in Dsi-PBX2 transfected cells and viceversa) (Fig. 5*e* and Supporting Information Fig. S1d).

Notably, treatment of both Mel888 and A375M with the HXR9 peptide also led to a clear down-regulation of both miR-221 and -222 in comparison with CXR9-treated cells (Fig. 5*f*), once again confirming the higher affinity of the HOXB7/PBX2 complex for DNA and their requirement for miR-221&222 transcription.

In view of this signaling, we tried to determine whether miR-221&222 per se were involved in the apoptotic process. As we already reported^18^ and extended here, miR-221&222 are gradually upregulated during melanoma progression and their expression levels associated with increased proliferation (Supporting Information Fig. S3). We then selected the A375M metastatic cell line for evaluating the functional effects deriving from miR-221&222 inhibitions by transfecting the antagomiR-221 and/or -222 molecules. The evaluation of the percentage of apoptotic cells by cytofluorimetric analysis of propidium iodide incorporation and Annexin V binding revealed an increase of ∼15% in cell death in treated cells with respect to cells either untreated or treated with an unrelated antagomiR (*i.e*., the antisense sequence targeting miR-133a, whose expression is totally absent in melanoma, our unpublished result) (Fig. 5*g*, left). The specificity of the down-regulation of these two miRs was confirmed by qRT-PCR (Fig. 5*g*, right).

**Figure 6:**
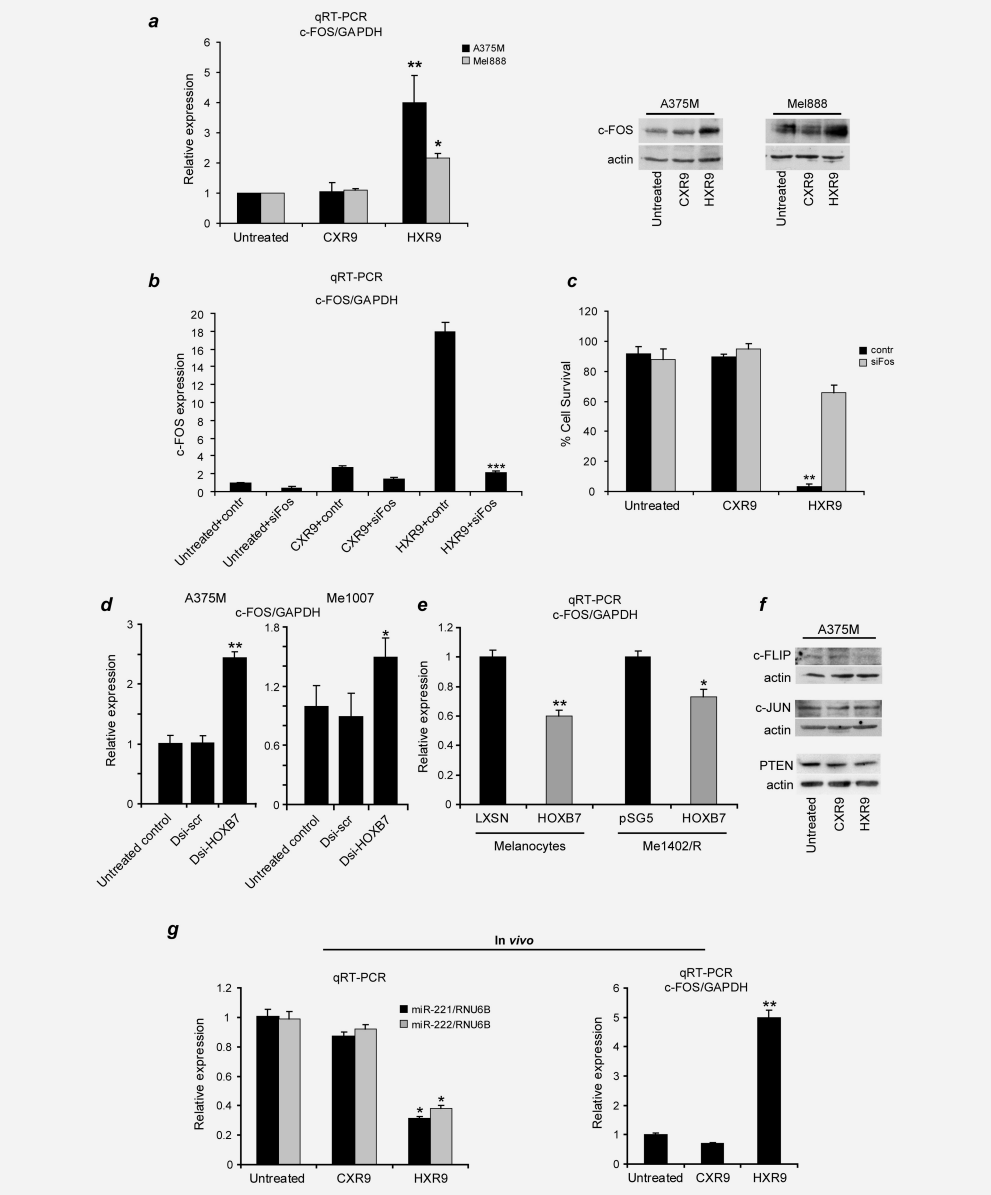
c-FOS regulation in HXR9-treated cells. (*a*) qRT-PCR (*left*) and representative WB (*righ*t) of c-FOS. (*b*) *c-FOS* expression and (*c*) evaluation of cell survival in A375M cells treated with an anti-c-FOS siRNA (siFOS) or a control siRNA (contr) 24 hr before treatment with 40 µM HXR9 or CXR9. Evaluation of *c-FOS* level by qRT-PCR in (*d*) A375M and Me1007 cells after HOXB7 silencing and in (*e*) HOXB7-overexpressing normal human melanocytes and Me1402/R melanoma cell line. LXSN and pSG5 are the empty vector controls. (*f*) Representative WB of c-FLIP, c-JUN and PTEN in the A375M melanoma cell line treated with 40 μM CXR9 or HXR9. Actin was the internal loading control. (*g*) qRT-PCR of miR-221, miR-222 and c-FOS in RNAs extracted from tumors recovered from mice 21 days after s.c. injection of A375M melanoma cells. Actin, *RNU6B* and *GAPDH* were used for normalization. *p < 0.05, **p <0.01, ***p < 0.001.

As qRT-PCR (Fig. 6*a*, left) and western blot analyses (Fig. 6*a*, right) confirmed c-FOS induction in HXR9-treated melanoma cells, we tested whether this up-regulation might be directly responsible for the HXR9-mediated apoptosis. A375M cells were transfected with a siRNA specifically targeting *c-FOS* mRNA (siFOS) prior to HXR9 treatment. A second non-targeting siRNA was used as a control (contr) and real-time qRT-PCR analysis confirmed the specific down-regulation of *c-FOS* (Fig. 6*b*). This reduction resulted in a significant protection from HXR9-induced cell death as shown by the increased percentage of surviving cells (from 10 to 65%) in presence of siFOS (Fig. 6*c*).

To definitely support the existence of a link among HOXB7, PBX2 and c-FOS, we analyzed *c-FOS* mRNA in Dsi-RNA-mediated silencing of either HOXB7 or PBX2: a statistically significant induction of c-FOS (approximately 2.5- and 1.5-fold) was obtained in both silenced conditions compared with control A375M cells (Fig. 6*d* and Supporting Information Fig. S1e). Accordingly, HOXB7 overexpression induced a decrease of *c-FOS* level (Fig. 6*e*).

As the c-FOS/c-JUN heterodimers have been shown to repress the antiapoptotic molecule c-FLIP(L),^26^ we decided to compare also c-JUN and c-FLIP(L) protein levels in untreated, CXR9- or HXR9-treated A375M. We did not observe any c-JUN modulation, whereas the c-FLIP(L) protein level was reduced in HXR9-treated cells (Fig. 6*f*). In addition, western blot analysis of the tumor suppressor protein PTEN, reported as an additional target of miR-221&222 in NSCLC,^17^ did not show any significant modulation after HXR9 treatment in melanoma cells (Fig. 6*f*).

Expression studies obtained by qRT-PCR on day 21 tumor nodules, confirmed a HXR9-based down-regulation of miR-221 and -222 followed by *c-FOS* restored expression also *in vivo* (Fig. 6*g*), further supporting the option of *c-FOS* as a direct target of miR-221&222.

Finally, the effectiveness of this regulatory pathway was assessed in bioptic samples from patients by immunohystochemistry for HOXB7 and c-FOS (Supporting Information Fig. S4a) and in situ hybridization for miR-221&222 (Supporting Information Fig. S4b). Results confirmed lower HOXB7 and higher c-FOS in primary respect to advanced melanomas (Supporting Information Fig. S4a). Accordingly, miR-221&222 direct and inverse correlations with HOXB7 and c-FOS are shown (Supporting Information Fig. S4b).

## Discussion

Melanoma is an aggressive malignancy, the incidence of which is increasing very rapidly worldwide. Although it is a highly curable disease in its early stages, advanced diseases lack effective treatments.^1^ An improved understanding of the underlying mechanisms is important in order to identify novel therapeutic targets.

Homeobox (HOX)-containing genes represent a subset of transcription factors involved in normal development and organogenesis.^3^ *HOX* gene clusters are a paradigm of genetic redundancy deriving from genome duplications. Nonetheless HOX proteins display a high regulatory specificity possibly through their cooperation with cofactors and other transcription factors.^9^ In melanoma we demonstrated that *HOXB7* participates in the autocrine regulation of bFGF through direct transactivation. *HOXB7* expression appeared tightly regulated in normal human melanocytes and constitutively activated in melanomas.^8^ In addition Wu and coauthors demonstrated that breast cancer cell lines, transfected with *HOXB7* start to display many features related to EMT, such as loss of expression of adhesion molecules and changes in cell morphology and cytoskeletal arrangement.^27^ Concordantly, other recent findings suggested *HOXB7* could be a valuable prognostic factor in colorectal cancer^28^ and oral squamous cell carcinoma.^29^

Our previous studies also demonstrated the requirement of Three Amino acid Loop Extension (TALE) cofactors for HOXB7 associated oncogenesis and the ability of HOXB7 to regulate this class of molecules, increasing the expression of PBX2 and reducing that of PBX1.^9^ Consistently, we showed that the SkBr3 breast carcinoma cell line, upon transduction of *HOXB7*, became positive for bFGF acquiring a more malignant phenotype.^8^ In the same cells we found a HOXB7-dependent induction of several proliferative and pro-angiogenic molecules such as VEGF, GROa, ANG-2 and IL-8.^7^ In this study we have shown that there is a striking HOXB7-dependent upregulation of miR-221&222, whose tumorigenic functions have been described in several type of cancer.^18^,^30^,^31^ In particular, in melanoma they enhance tumorigenicity by targeting, among many other molecules, p27^Kip1^, ETS-1 and c-KIT receptor, thus leading to enhanced proliferation, dissemination and differentiation blockade of melanoma cells.^18^,^21^

Few data do exist on microRNA transcriptional regulation. For miR-221&222, we had already identified the promyelocytic leukemia zinc finger (PLZF), previously reported as a tumor suppressor down-modulated in melanomas,^32^ as an upstream negative regulator of miR-221 and miR-222. In addition, we demonstrated a miR-221&222/ETS1 circuitry where ETS1, besides being targeted by miR-222, plays a transcriptional activating role on miR-221&222 according to its ERK-dependent phosphorylation status.^21^ Here we show that the HOXB7/PBX2 complex is a direct transcriptional activator of miR-221&222. By using HXR9, an antagonist of HOX/PBX dimer formation, we confirmed the requirement of these complexes for effective binding to DNA and transcription. HXR9 treatment results in the downregulation of miR-221&222 in human melanoma cells as a consequence of HOXB7/PBX2 heterodimer disruption and the subsequent degradation of HOXB7 and PBX2 proteins. This apparent instability of HOX and PBX monomers may reflect a regulatory mechanism similar to that reported for the C/EBP family of proteins, whereby monomers are degraded to avoid any intracellular unbalance, and the proteins are stabilized by either homo- or heterodimerization.^33^ Results reporting treatments with the proteasome inhibitor MG132 showed a cytoplasmic accumulation of PBX2, suggesting that its degradation goes through the proteasome, particularly when it is in a monomeric excess over its partners.^34^ Also a Mass Spectrometry analysis, performed on human lung adenocarcinoma cells (H1299), suggested HOXB7 as a substrate of the ubiquitination machinery.^35^ In melanoma cells HOXB7 silencing confirmed the lower stability of PBX2 when the lack of its partner hampers a correct dimerization and *vice versa* (Fig. 5*e* and Supporting Information Fig. S1d).

In this study we have identified *c-FOS* as an additional target of miR-221&222 in melanoma, describing the functionality of a HOX/PBX→miR-221&222→c-FOS pathway. C-FOS, together with c-JUN, is part of the activator protein-1 (AP-1), a complex able to exert either pro- or anti-apoptotic functions in different cell populations, including tumor cells.^36^ Increased *c-FOS* has been associated with apoptosis in different cell types subjected to various treatments,^12^–^15^ including TRAIL-induced apoptosis through c-FLIP(L) repression in prostate cancer.^26^,^37^ As previously reported by Croce and collaborators for non-small cell lung cancer (NSCLC), c-JUN, but not c-FOS, is able to transactivate miR-221&222 by direct binding to their promoter region.^16^ In HXR9-induced apoptosis in melanoma, we found miR-221&222 downregulation directly associated with c-FOS upregulation, thus confirming the specificity of miR-221&222/c-FOS, with no effects on the other member of the AP-1 complex. We also reported the lack of any evident involvement of PTEN in HXR9-induced apoptosis. Although PTEN has been validated as a miR-221&222-dependent target in HCC and NSCLC,^17^ it does not seem to exert this function in HXR9-treated melanomas, thus strengthening the key role of c-FOS in inducing apoptosis.

Surprisingly, the analysis of miR-221&222 and c-FOS levels in the panel of melanoma cell lines did not reveal the expected obvious inverse correlation between microRNAs and their targets (Fig. 3*d*). However, c-FOS protein is stabilized by post-translational modifications, such as phosphorylation at serines 362 and 374,^22^–^24^ whose levels are directly related with the activation of the MAPK pathway.^38^ In view of that, c-FOS levels in the analyzed cell lines, besides being regulated by miR-221&222, are dependent upon the phosphorylation status of ERK1/2, as suggested for the NGF-dependent cell survival in PC12 cells.^39^ This might explain the low level of c-FOS detectable in primary melanomas (as Mel501 and Me1007) lacking any of the frequent mutations in B-RAF or N-RAS genes. These melanomas, although expressing low levels of miR-221&222, display a small amount of c-FOS according to its barely phosphorylated status. Accordingly, BRAF^V600E^ transfection in Me1007 melanoma induced a striking ERK1/2 activation followed by c-FOS induction (Fig. 3*e*). Thus, the reduced expression of c-FOS in metastatic melanomas would be expected to depend upon the repressive function of miR-221&222 which appear to overcome c-FOS stability.^18^ It is finally important to point out the extreme effectiveness of HXR9 in the induction of apoptosis in human melanoma cells and the absence of any HXR9-derived toxicity in the normal counterpart, here represented by primary human melanocytes. Accordingly, a lack of any apparent toxicity has been shown in HXR9-treated mice. In particular, no abnormalities have been detected in hematopoietic cells, either from bone marrow or peripheral blood, where the HOX genes are known to be highly functional.^12^

Taken together, our results suggest that the direct inhibition of miR-221&222 by antagomiR treatment and/or the disruption of the HOXB7/PBX2 dimers might represent an innovative approach for translation into the clinical setting (Supporting Information Fig. S5). Furthermore, as this and other studies suggest the existence of common regulatory signaling for miR-221&222 and HOXB7,^18^,^40^,^41^ the combined treatments could be more effective due to a synergistic interaction.
