# Supplementary material for: The abrogation of the HOXB7/PBX2 complex induces apoptosis in melanoma through the miR-221&222-c-FOS pathway
Source: Int J Cancer. 2013 Feb 7;133(4):879–92. doi: 10.1002/ijc.28097 (PMC3812682; doi:10.1002/ijc.28097)
Supplement: Supplementary file 1 [file ijc0133-0879-SD1.pdf]

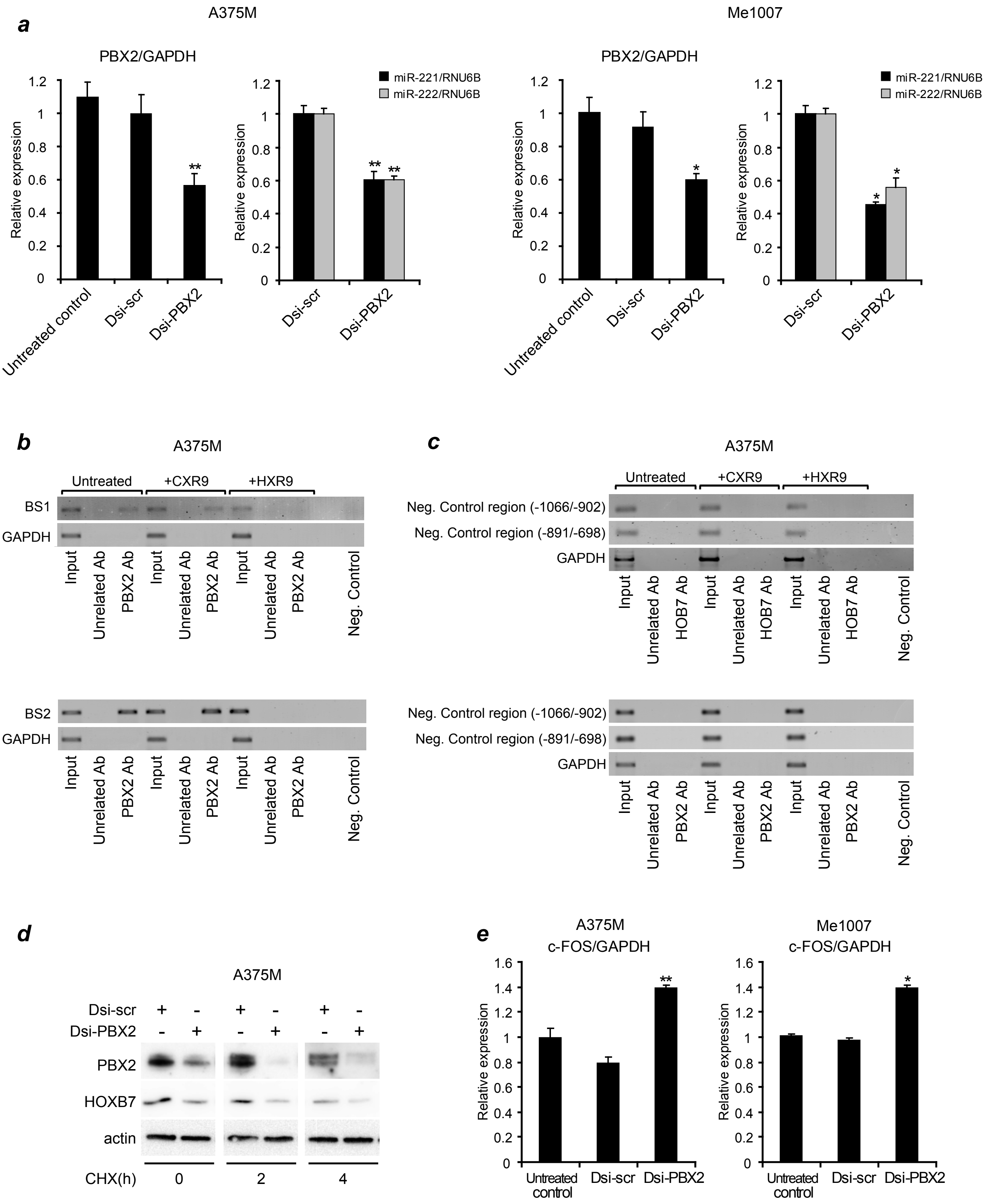

Supplementary Fig. S1. *(a)* qRT-PCR evaluation of *PBX2* and miR-221&222 in Dsi-PBX2-transfected A375M *(left)* and Me1007 *(right)* cell lines. *(b)* Chromatin immunoprecipitation assays performed in A375M cells treated with 40 µmol/L of HXR9 or CXR9. *(c)* The binding specificity and the absence of other HOX/PBX binding sites was confirmed through the analysis of two negative control regions, (-891/-698) and (-1066/-902). *(d)* Evaluation of PBX2 and HOXB7 stabilities in A375M cells transfected with either a Dsi-scrambled control (Dsi-scr) or with Dsi-PBX2 and treated with cycloheximide (CHX) up to 4 h. *(e) c-FOS* level analyzed by qRT-PCR in A375M *(left)* and Me1007 *(right)* cells after *PBX2* silencing. Actin, *RNU6B* and *GAPDH* were used for normalization. * $p<0.05$, ** $p<0.01$.
