# Supplementary material for: The abrogation of the HOXB7/PBX2 complex induces apoptosis in melanoma through the miR-221&222-c-FOS pathway
Source: Int J Cancer. 2013 Feb 7;133(4):879–92. doi: 10.1002/ijc.28097 (PMC3812682; doi:10.1002/ijc.28097)
Supplement: Supplementary file 2 [file ijc0133-0879-SD2.pdf]

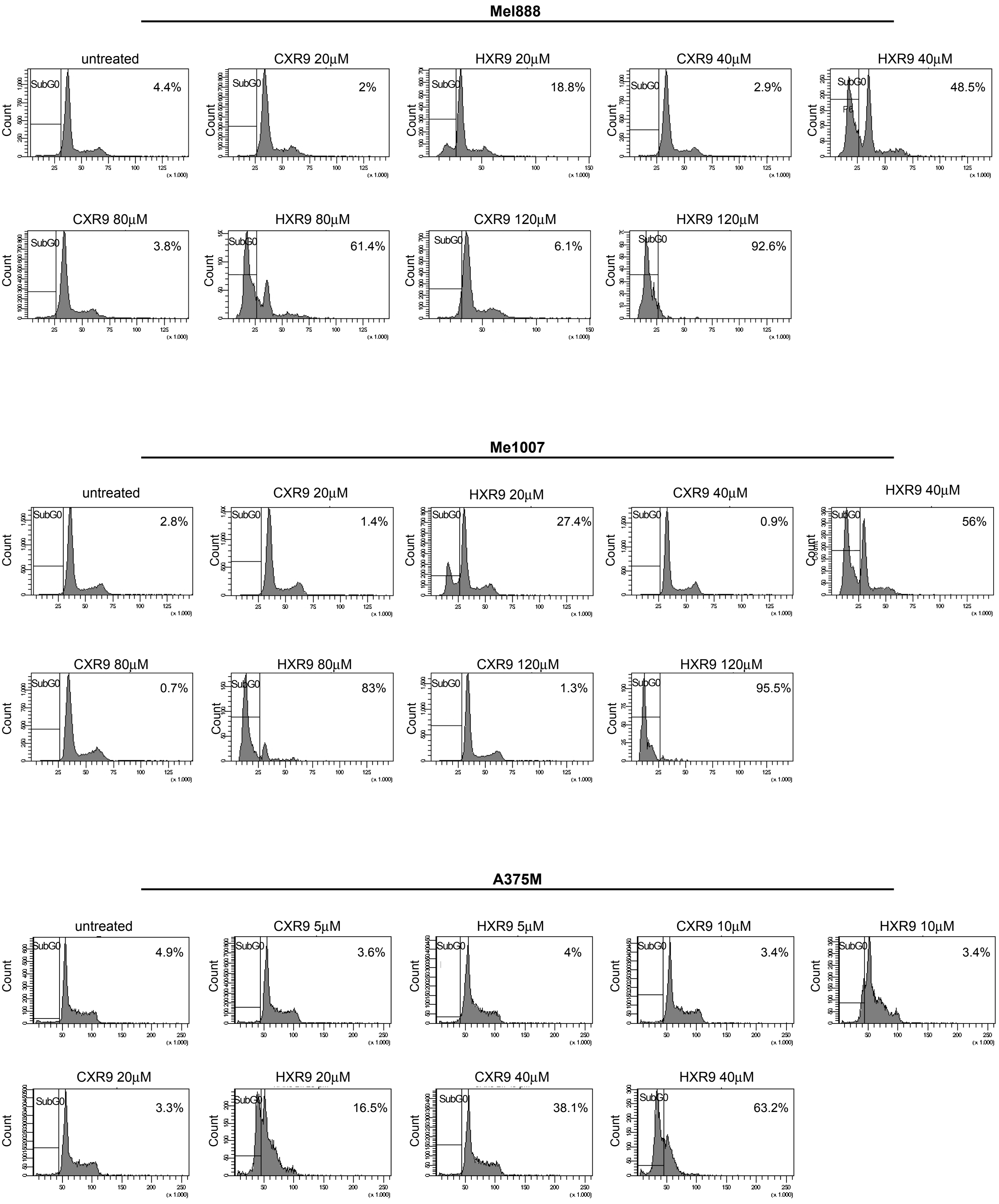

Supplementary Fig. S2. Representative dose-response results deriving from CXR9- or HXR9-treatments of Mel888, Me1007 and A375M cell lines. The percentage values indicate the apoptotic SubG0 cell population.
