# Supplementary material for: The abrogation of the HOXB7/PBX2 complex induces apoptosis in melanoma through the miR-221&222-c-FOS pathway
Source: Int J Cancer. 2013 Feb 7;133(4):879–92. doi: 10.1002/ijc.28097 (PMC3812682; doi:10.1002/ijc.28097)
Supplement: Supplementary file 3 [file ijc0133-0879-SD3.pdf]

a

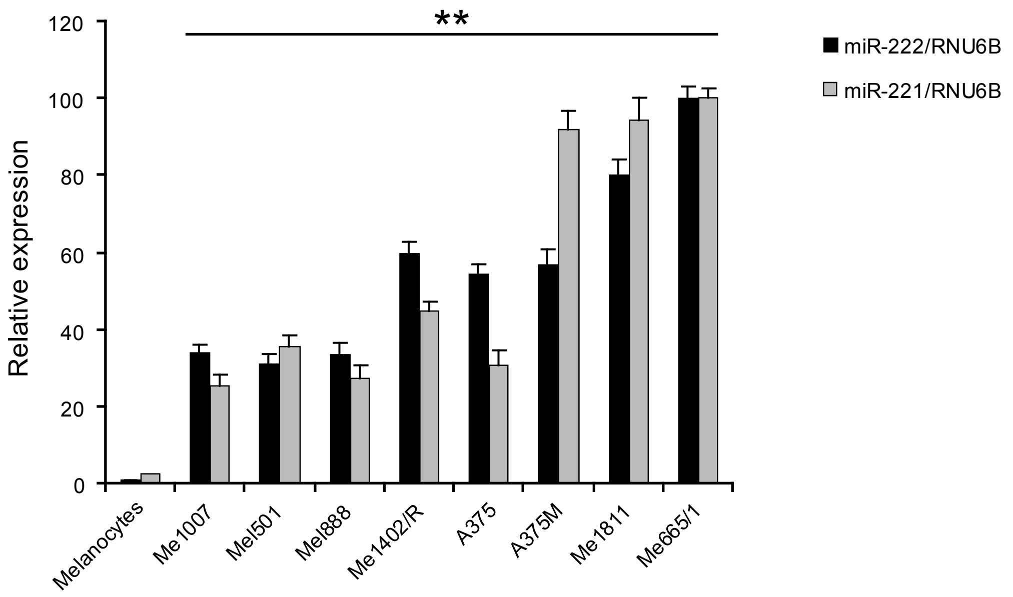

b

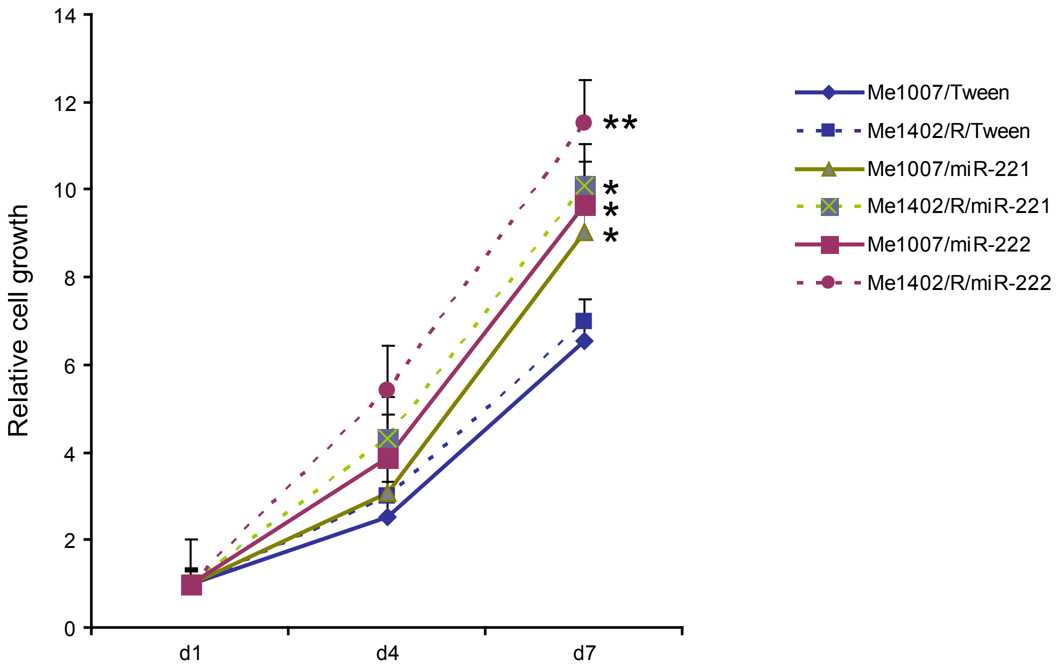

Supplementary Fig. S3 MiR-221 and miR-222 expression in normal human melanocytes and melanoma cell lines. *(a)* Relative expression values obtained by qRT-PCR. *RNU6B* was used as internal control. *(b)* Cell growth proliferation analysis in Me1007 and Me1402/R cell lines transduced with miR-221 or mir-222 and compared with control vector (Tween). *p <0.01, **p<0.05.
