# Supplementary material for: The abrogation of the HOXB7/PBX2 complex induces apoptosis in melanoma through the miR-221&222-c-FOS pathway
Source: Int J Cancer. 2013 Feb 7;133(4):879–92. doi: 10.1002/ijc.28097 (PMC3812682; doi:10.1002/ijc.28097)
Supplement: Supplementary file 4 [file ijc0133-0879-SD4.pdf]

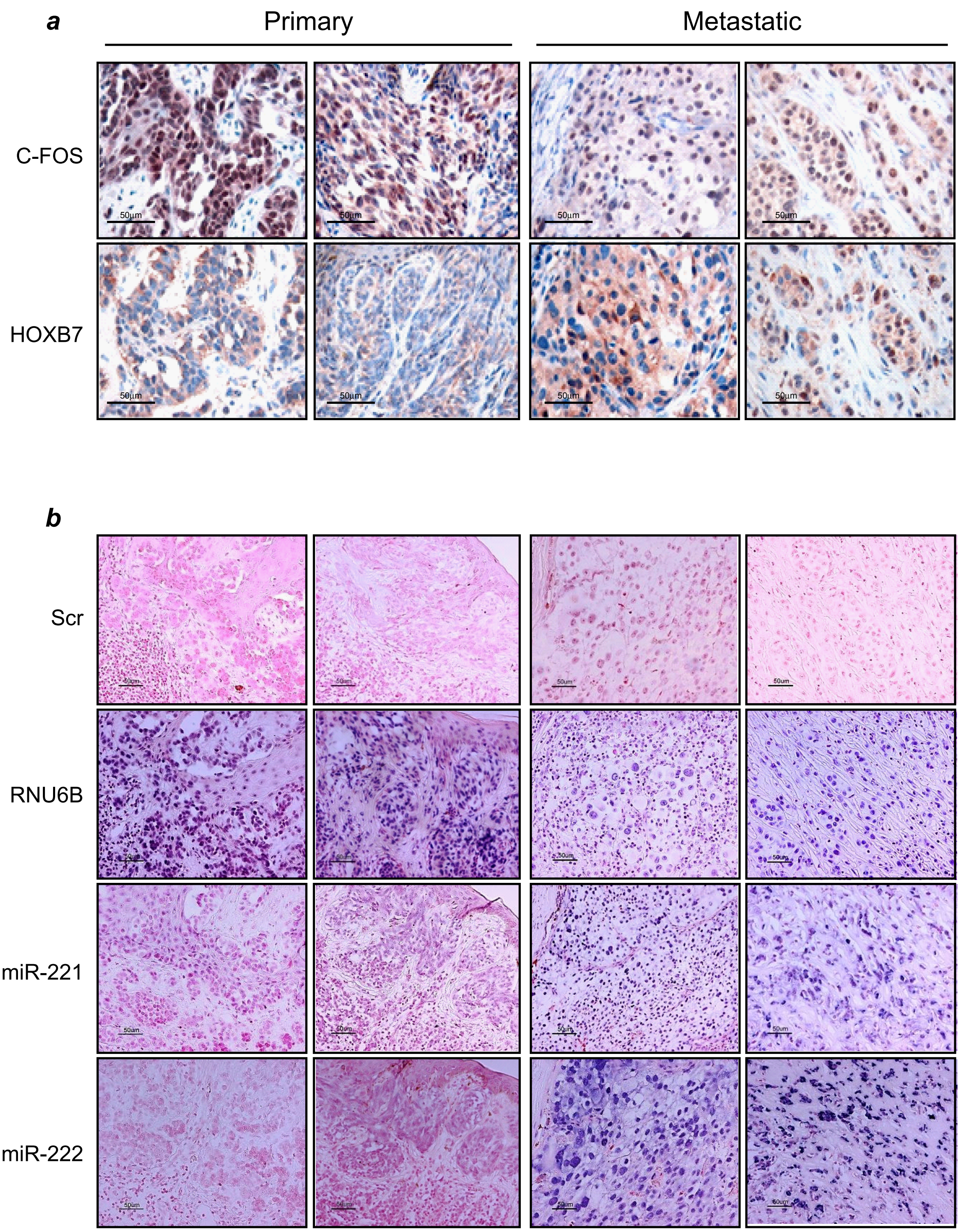

Supplementary Fig. S4. *(a)* Immunohistochemical analysis on primary and metastatic melanoma, displaying the inverse correlation between c-FOS and HOXB7 expression. Four representative immunostained sections (2 primary and 2 metastatic melanomas) are shown (magnification x400) . *(b)* In situ hybridization of miR-221 and miR-222 on sections from the same melanoma specimens indicating, respectively, their inverse and direct correlation with c-FOS and HOXB7 expressions (magnification x200). Scr and RNU6B correspond to the negative and positive controls.
