# Supplementary material for: The abrogation of the HOXB7/PBX2 complex induces apoptosis in melanoma through the miR-221&222-c-FOS pathway
Source: Int J Cancer. 2013 Feb 7;133(4):879–92. doi: 10.1002/ijc.28097 (PMC3812682; doi:10.1002/ijc.28097)
Supplement: Supplementary file 5 [file ijc0133-0879-SD5.pdf]

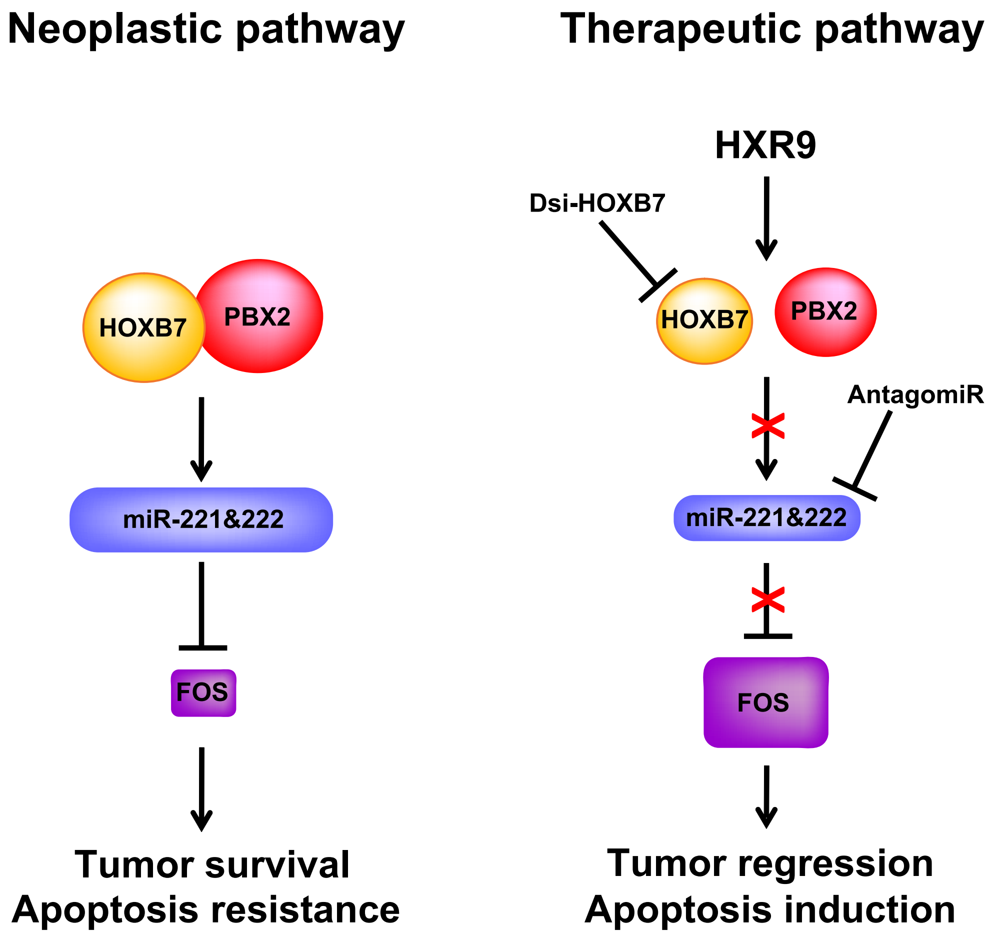

Supplementary Fig. S5. Schematic description of HOXB7/PBX2 dependent activation of miR-221&222 and c-FOS targeted downregulation in melanoma *(left)*; according to hypothetical therapeutic approaches, HXR9-dependent disruption of HOXB7/PBX2, HOXB7 and/or miR-221&222 repression might reduce melanoma malignancy *(right)*.
