# Supplementary material for: The abrogation of the HOXB7/PBX2 complex induces apoptosis in melanoma through the miR-221&222-c-FOS pathway
Source: Int J Cancer. 2013 Feb 7;133(4):879–92. doi: 10.1002/ijc.28097 (PMC3812682; doi:10.1002/ijc.28097)
Supplement: Supplementary file 6 [file ijc0133-0879-SD6.docx]

**Data Supplement:**

• **Supplementary Materials and Methods**

• **Supplementary Table 1**

• **Supplementary References**

**Supplementary Materials and Methods**

**RNA extraction and qRT-PCR.** Total RNA was extracted with NucleoSpin® miRNA kit (Macherey-Nagel) according to the manufacturer’s specifications. In order to accurately detect mature miR-221 and -222, a Real Time quantification (qRT-PCR) method was performed according to the TaqMan MicroRNA Assays from Applied Biosystems (miR-221#000524; miR-222 # 000525). qRT-PCR analysis of c-FOS (#Hs04194186), HOXB7 (#Hs04187556) and PBX2 (#Hs01901345) was performed by TaqMan gene expression tecnology assay from Applied Biosystems (Applied Biosystems, Foster City, CA). Samples were normalized by evaluating RNU6B (#001093) and GAPDH (#Hs02758991) expression.

**Western blot** was performed according to standard procedures. Cell lysates were separated by the precast NuPAGE polyacrilamide gel system (Invitrogen, Camarillo, CA). Antibodies against HOXB7 (Invitrogen, Camarillo, CA), PBX2 and C-JUN (Santa Cruz Biotechnology, Santa Cruz, CA), c-FOS, ERK1⁄2 and P-ERK1⁄2 (Cell Signaling Technology Danvers, MA), PTEN (BD Pharmingen™ San Diego, CA) and c-FLIP (Enzo Life Sciences, Lausen, Switzerland) were used in accordance to the manufacturer’s instructions. ACTIN (OncogeneResearch Products, Boston, MA) was used as a loading control. The expression levels were evaluated by the AlphaView Software.

**Cell growth analysis.** The proliferative rate of melanoma cells was evaluated by a colorimetric assay XTT-based (Roche Molecular Biochemicals, Mannheim, Germany) and quantified using an ELISA plate reader (VICTOR2, Perkin Elmer).

**Caspase-3 Activity.** Caspase assays were performed EnzChek® Caspase-3 Assay Kit 2 according to manufacturer instructions. The caspase-3 activity in the supernatant was analyzed spectrophotometrically in a Beckman Coulter DTX 880 Multimode Detector using excitation at 485 nm and emission at 535 nm (caspase activity in the cell lysate leads to the cleavage of the non-florescent substrate into a florescent product). The specificity of the caspase 3 activity was determined by the addition of a caspase-3 inhibitor (Ac-DEVD-CHO Inhibitor).

**Co-immunoprecipitation.** Protein A Dynabeads (Invitrogen, Camarillo, CA) were prepared according to the manufacturer's directions. Briefly, anti-HOXB7 antibody (2 µg) was incubated with protein A Dynabeads in 50 µl Sodium Phosphate Buffer 0.1 M pH 8.1, with gentle shaking for 10 minutes at room temperature. The cross-linking was then achieved resuspending the antibody/beads mixture in a buffer containing TEA (triethanolamine) 0.2M pH 8.2 and DMP (Dimethyl pimelimidate dihydrochloride) 20mM (Pierce) with gentle shaking for 30 minutes at room temperature. Protein lysates were then added to these mixtures and immunoprecipitation performed at 4°C for one hour. Following the final wash with 1X PBS, beads were resuspended in NuPAGE SDS-PAGE buffer, heated at 95°C for 10 minutes, and the supernatants loaded onto 10% Bis-Tris NuPAGE gels. Blots were then probed with anti-PBX2 antibody.

**Target analysis**. Bioinformatic analysis was performed by using these specific programs: TargetScan (<http://www.targetscan.org/>) and RNAhybrid (<http://bibiservice.techfak.uni-bielefeld.de/>).

**Luciferase assay.** In order to analyze the functional roles of the putative HOX/PBX binding site TAATTGAT (indicated as BS1 and BS2 in Fig. 2E), two DNA fragment containing the putative regulatory region upstream to miR-222&221 (from -352 to +1 nt and from -1786/-1035 nt) was amplified and cloned in pGL3 basic (Promega, Madison, WI). A375M cell line was transfected with Fugene HD (Promega, Madison, WI) and: (a) 200 ng of pGL3 basic or pGL3 containing the above genomic fragment, (b) 100 nM of Dsi-scr or Dsi-HOXB7 and (c) 20 ng of Renilla. The pGL3 plasmid, containing the region from -352 to +1 nt and cotransfected with the Dsi-scr was considered as 100%. As controls of specificity, point mutations were inserted in the wild type core binding sequence for HOXB7/PBX by using the QuickChange site-directed mutagenesis kit (Stratagene).The sequences of the oligonucleotides are listed below; bold capital letters indicate the core sequences, and lowercase letters indicate the mutated base:

BS1 (wild-type) 5’-AACATACCAAA**TAATTGATTG**TAGATTTTG-3’.

BS1 (mutated) 5’-AACATACCAAA**gAgcTcgTTG**TAGATTTTG-3’

BS2 (wild-type) 5’-AAATTAGACATCTG**TGATTA**AATAGTTAAA -3’.

BS2 (mutated) 5’-AAATTAGACTTCT**GcagTgA**AATAGTTAAA-3’.

For the luciferase reporter experiments, a 372-bp fragment of the c-FOS 3’ UTR containing the predicted miR-221 and -222 conserved putative binding site was amplified by PCR from normal human genomic DNA using a JumpStart™ AccuTaq™ LA DNA Polymerase (Sigma, St. Louis, MO) using primers fwd 5’ACAAGTGCCACTGCCCGA-3’ and rev 5’-TGTATCTAGTGCAGCTGA-3’. The putative c-FOS-1 seed starts at nt 1551 of c-FOS sequence (NCBI Reference Sequence: NM_005252.3). After sequence analysis, the construct was subcloned into the pGL3 promoter vector (Promega, Madison, WI), immediately downstream from the stop codon of the luciferase gene. The putative c-FOS seed and its mutated version were 5’ TTACCTCTTCCGGAG**ATGTAGC**A 3’ and 5**’** TTACCTCTTCCGGAG**AgcTcGC**A 3’, respectively. The conserved core is indicated in bold while lower case letters represent the mutated nucleotides. 293FT cells (5x10^4^ cells per well) were transfected with: (a) 20 ng of pGL3-3’ UTR plasmid, (b) 15 pmol of either a stability-enhanced 2’-O-Methyl non targeting RNA control or miR-221 and/or miR-222 oligonucleotides (Dharmacon Inc. Pittsburgh PA), (c) Fugene HD (Promega, Madison, WI) and (d) 10 ng of Renilla. The wt pGL3-3’UTR cotransfected with the control non targeting oligonucleotide was considered as 100%.

In all the experiments the luciferase activity was measured after 48h by using the FemtomasterFB 12 (Zylux). Ratios between Firefly and Renilla luciferase activities were measured with a dual luciferase assay (Promega, Madison, WI).

**Immunohistochemical Staining.** For in situ single-marker immunohistochemical analysis sections were deparaffinized with xylene and rehydrated to water through a graded alcohol series. Antigen unmasking was performed using a microwave epitope retrieval technique with 10 mmol citrate buffer (pH 9.0) at high temperature for 20 min. Endogenous peroxidase activity was quenched with 3% hydrogen peroxide. Sections were incubated with anti-human c-FOS or anti-human HOXB7 (Abcam, Cambridge, UK) at room temperature for 1 hour. Staining was performed with the Novolink Max Polymer Detection System (Leica Microsystems) and with AEC (3-amino-9-ethylcarbazole) substrate chromogen (Dako Italia, Milano, ITALY). Finally, sections were countestained with hematoxilyn. Slides were evaluated using a Leica DM2000 equipped with a Leica DFC320 digital camera.

**In situ miRNA hybridization.** In situ detection of miR-221-222 on formalin fixed paraffin embedded melanoma samples was essentially performed as described by miRCURY Locked-nucleic Acids (LNA^TM^s) ISH optimization kit from Exiqon (Exiqon, Vedbaek, Denmark) (*6*). 5’-3’Double-DIG labeled complementary miR-221, miR-222 and 5’DIG labeled RNU6B (control) and scrambled (negative control) oligonucleotides were used as probes. Tissue sections were incubated with diluted probes (40 nM for miRs and 2 nM for RNU6B and scrambled probes) for 1 hour at a temperature of 30°C below the calculated melting temperature of the probe. After post hybridization washes at hybridization temperatures, bound probes were detected by enzyme coupled antibodies (AP conjugated anti-Digoxigenin Fab fragments from Roche Mannheim, Germany) and a subsequent color reaction using the NBT/BCIP reagent (Roche) was executed. Tissue sections were counterstained with nuclear fast red staining solution (Sigma, Deisenhofen, Germany). Microscopy analysis was performed with a Nikon Eclipse E 1000 Microscope equipped with a Nikon Dxm 1200 digital camera. A Nikon ACT-1 program was used for image acquisition (Nikon).

**Supplementary Table 1**

| **Table 1** |  |  |
| --- | --- | --- |
| **Melanoma cell lines analyzed in this study** | |  |
| **Cell line** | **Type** | **Reference** |
| **Mel501** | **Primary tumor** | **1** |
| **Mel888** | **Primary tumor** | **2** |
| **Me1007** | **Primary tumor** | **3** |
| **Me1402/R** | **Recurrence of primary tumor** | **3** |
| **A375** | **Metastatic melanoma** | **4** |
| **A375M** | **Metastatic melanoma** | **5** |
| **Me1811** | **Lymph node metastasis** | **3** |
| **Me665/1** | **Lymph node metastasis** | **3** |
|  |  |  |

**Supplementary References**

1. Supino R, Mapelli E, Sanfilippo O, Silvestro L, Biological and enzymatic features of human melanoma clones with different invasive potential. Melanoma Res 1992; 2:377-384.
2. Alexaki VI, Javelaud D, Van Kempen LC, Mohammad KS, Dennler S, Luciani F, et al.: GLI2 Mediated Melanoma Invasion and Metastasis. J Natl Cancer Inst 2010; 102:1148-59.
3. Colombo MP, Maccalli C, Mattei S, Melani C, Radrizzani M, Parmiani G, Expression of cytokine genes, including IL-6, in human malignant melanoma cell line. Melanoma Res 1992; 2:181-189.
4. [Giard DJ](http://www.ncbi.nlm.nih.gov/pubmed?term=%22Giard%20DJ%22%5BAuthor%5D), [Aaronson SA](http://www.ncbi.nlm.nih.gov/pubmed?term=%22Aaronson%20SA%22%5BAuthor%5D), [Todaro GJ](http://www.ncbi.nlm.nih.gov/pubmed?term=%22Todaro%20GJ%22%5BAuthor%5D), [Arnstein P](http://www.ncbi.nlm.nih.gov/pubmed?term=%22Arnstein%20P%22%5BAuthor%5D), [Kersey JH](http://www.ncbi.nlm.nih.gov/pubmed?term=%22Kersey%20JH%22%5BAuthor%5D), [Dosik H](http://www.ncbi.nlm.nih.gov/pubmed?term=%22Dosik%20H%22%5BAuthor%5D), et al. In vitro cultivation of human tumors: establishment of cell lines derived from a series of solid tumors. J Natl Cancer Inst. 1973, 51:1417-23.
5. Kozlowski JM, Hart IR, Fidler IJ, Hanna N. A human melanoma line heterogeneous with respect to metastatic capacity in athymic nude mice. J Natl Cancer Inst 1984, 72:913-7.
6. Jørgensen S, Baker A, Møller S, Nielsen BS. [Robust one-day in situ hybridization protocol for detection of microRNAs in paraffin samples using LNA probes.](http://www.ncbi.nlm.nih.gov/pubmed/20621190) Methods. 2010; 52(4):375-81.
